# Optical Coherence Tomography, Stereomicroscopic, and Histological Aspects of Bone Regeneration on Rat Calvaria in the Presence of Bovine Xenograft or Titanium-Reinforced Hydroxyapatite

**DOI:** 10.3390/jfb17010026

**Published:** 2026-01-01

**Authors:** Andrei Radu, Antonia Samia Khaddour, Mihaela Ionescu, Cristina Maria Munteanu, Eugen Osiac, Oana Gîngu, Cristina Teișanu, Valentin Octavian Mateescu, Cristina Elena Andrei, Sanda Mihaela Popescu

**Affiliations:** 1 Department of Oral Rehabilitation, University of Medicine and Pharmacy of Craiova, 200349 Craiova, Romaniasanda.popescu@umfcv.ro (S.M.P.); 2 Department of Medical Informatics and Biostatistics, University of Medicine and Pharmacy of Craiova, 200349 Craiova, Romania; 3 Department of Oral and Maxillofacial Surgery, University of Medicine and Pharmacy of Craiova, 200349 Craiova, Romania; 4 Department of Biophysics, University of Medicine and Pharmacy of Craiova, 200349 Craiova, Romania; 5 Department of Engineering and Management of Technological Systems, Faculty of Mechanics, University of Craiova, 200585 Craiova, Romania; 6 Department of Histology, University of Medicine and Pharmacy of Craiova, 200349 Craiova, Romania

**Keywords:** bone graft, bone regeneration, biomaterial, bovine xenograft, hydroxyapatite, rat calvaria, OCT, histological analysis, stereomicroscopic analysis

## Abstract

Background: Alveolar ridge preservation (ARP) techniques have evolved with implantology development. In clinical practice, biomaterials for ARP are tested in laboratory animals, and rat calvaria is a standard option. The study aimed to evaluate biomaterial osteointegration in defects created in the rat calvaria, comparing an experimental synthetic biomaterial with a bovine xenograft and natural healing. Methods: The study included six groups of animals: two negative control groups with natural healing (2 months (M) and 4 M), two positive control groups with bovine xenograft (2 M and 4 M), and two study groups with nanohydroxyapatite titanium reinforced (2M and 4M). After creating and grafting the defects, healing was expected to take 2 or 4 months, after which bone fragments were harvested, prepared, and then analyzed. OCT, stereomicroscopy, and histology techniques were used for bone fragments analysis, and the obtained images were evaluated using Image J 1.54p software. Results: The results obtained from the three analyses provided information about the healing pattern of bone defects and the degree of new bone formation. Histological analysis of the samples confirmed what the stereomicroscopy and OCT images showed: that the bovine xenograft elicited a better tissue response than the synthetic biomaterial, being incorporated into the bone tissue more than the synthetic biomaterial. Conclusions: Both the bovine xenograft and the synthetic nanocomposite based on hydroxyapatite reinforced with titanium particles favored bone healing, but their integration into the bone was limited for the analyzed period.

## 1. Introduction

With the loss of dental units, a physiological process of three-dimensional alveolar bone resorption begins, which limits the long-term success of the implant-prosthetic treatment plan [1,2,3,4]. The evolution of implantology towards a conventional therapy option has also driven increased interest in the development of post-extraction alveolar ridge preservation (ARP) techniques. As a result, the different types of biomaterials indicated for these procedures have been increasingly studied in the literature [4,5,6] to obtain a resistant, sufficiently voluminous bone structure to support the implant [7,8]. ARP reduces bone loss after tooth extraction, thereby improving implant-prosthetic outcomes [9].

A wide range of biomaterials is used for bone defect regeneration [10,11,12,13,14]. The ideal material for bone defect augmentation possesses properties similar to those of natural bone [15]. Biomaterials used for post-extraction alveolar ridge preservation are divided into four major categories (autografts, allografts, xenografts, alloplasts), and are obtained from different sources (human, animal, synthetic) [6,16]. The ideal biomaterial must meet several criteria, including being biocompatible, supporting bone healing and reconstruction, and being resorbed and replaced by native bone [17] (osteoconductive, osteoinductive, osteogenic, and promoting osseointegration) [5,18]. Many authors have advocated autografts as the gold standard for bone regeneration [1,2,17,19,20,21]. These had a significant disadvantage: a second site of intervention—the graft harvesting area. Xenografts, especially those of bovine origin, are widely used in dentistry, especially in Europe [22,23,24,25]. Bovine xenografts have several advantages, including osteoconductivity, resistance to resorption, low risk of disease transmission, and a structural and chemical composition similar to human bone [26,27,28] and high structural stability [29]. The main disadvantage is their lower capacity for bone formation compared to autogenous grafts [30]. Another viable alternative to these types of bone grafts is synthetic materials [31,32]. Synthetic hydroxyapatite is a relatively chemically stable substance, remaining for a more extended period in the defect area. This fact constitutes both an advantage and a disadvantage, depending on the indication and the site of use [33]. The size and shape of biomaterial particles are essential for bone healing, influencing bone formation and mineralization [8,34,35,36]. Hydroxyapatite also has several advantages, including osteoconductive properties, chemical adhesion, a slow resorption rate, and structural stability [37,38,39,40,41,42]. It is biocompatible, with physical and chemical properties comparable to those of human bone [43]. The bovine xenograft is a porous bone mineral matrix, which allows rapid absorption of blood, nutrients, and growth factors. It can be compared to human bone due to its similar physical and chemical properties [44], but there are reservations regarding its osseointegration in the post-extraction socket [33].

To be used in humans, biomaterials must first be tested in laboratory animals [4,43,45]. In the literature, defects created in the rat calvaria are standard [3,32,45,46], and are rich in osteoblasts, which contribute to the formation of new bone tissue [47,48].

Optical coherence tomography (OCT) is a three-dimensional imaging technique that produces cross-sectional images of tissues and is very useful for studying hard and soft tissues [49]. It has high spatial resolution and significant contrast, allowing the study of osseointegration [50,51]. This analysis method uses low-power infrared (IR) laser radiation and can quantify changes in mineral tissues [49]. The advantages of the technique are its non-ionizing, non-invasive, and non-destructive nature, which allows the acquisition of two-dimensional images from three-dimensional samples [49,52,53]. The disadvantages of the method include the inability of light to penetrate to great depths, poor contrast in mineralized tissues, and the difficulty in volumetric analysis of regenerated tissues [52,54,55].

Stereomicroscopy is an analysis technique with a resolution suitable for evaluating the external morphology of tissues and offers the advantage of obtaining authentic three-dimensional images, with minimal sample preparation. Using stereomicroscopy, bone changes during bone regeneration can be observed by analyzing the external structure of the grafted biomaterials and the surrounding bone tissue [56,57].

Histological analysis is an optimal method for monitoring bone regeneration in animal studies, as it provides data on bone structure, resorption, and mineralization [58].

No studies using OCT, a stereomicroscope, and histological analysis were identified in the literature to compare bovine xenograft and synthetic biomaterial based on hydroxyapatite reinforced with titanium particles. Thus, the present study aimed to evaluate the degree of biocompatibility and the mode of osteoformation in defects created in the rat calvaria, using a bovine graft material and an experimental synthetic biomaterial, and to compare these with natural healing, as analyzed using OCT, stereomicroscopy, and histological images obtained from the study. The null hypothesis was that there were no differences in the healing pattern between the control and study groups.

## 2. Materials and Methods

### 2.1. Study Design

This study followed the ARRIVE Guidelines criteria. The experimental study analyzed the degree of bone formation in defects made on the calvaria of adult male Wistar laboratory rats, with natural healing or different grafted biomaterials. Rat calvaria is the experimental model currently considered the gold standard for histological evaluation of tissue-level changes, especially when using biomaterials not yet approved for human use. The study was conducted on adult laboratory animals because the potential for spontaneous bone repair is greater in young animals. The gender was male, as in female animals, hormonal changes influence the results [59].

The temporal component of the study consisted of evaluations at 2 and 4 months, which allowed assessment of changes produced over time around bone defects [59]. The experiment included laboratory animals within the Animal Unit of the University of Medicine and Pharmacy of Craiova. The Ethics Committee of the University of Medicine and Pharmacy of Craiova approved the working protocol (Approval number 231/28 November 2022).

### 2.2. Study Groups

A total of fifteen adult male Wistar rats, weighing between 250 g and 350 g (mean 300 g), were used for the experiment. Depending on the treatment of the bone defects, the groups were: the negative control group (natural healing), the positive control group (grafted with bovine bone), and the study group (grafted with synthetic bone) [60]. The results were analyzed in parallel, using the same methodology. The distribution of the animals into the initial three groups was random: group A (*n* = 5), negative control group (natural healing); group B (*n* = 5), positive control group (bovine xenograft); and group C (*n* = 5), the study group, to which an experimental nanocomposite biomaterial represented by hydroxyapatite reinforced with titanium-based particles was grafted. Laboratory animals were housed in standard cages, and food and water were provided ad libitum, according to the recommendations provided by the Veterinary and Food Safety Authority of Dolj County, Romania.

### 2.3. Biomaterials

Biomaterials used in the present study were a commercially available bovine xenograft and an experimental synthetic biomaterial based on hydroxyapatite reinforced with titanium-based particles. The bovine xenograft (Bio-Oss LOT 82300217, Geistlich Pharma AG, Zurich, Switzerland) is a porous, sterile, and biocompatible biomaterial that is physically and chemically comparable to the mineral structure of human bone matrix. It is available as spongy granules, and the variant used for the experiment had a volume of 0.5 g and a granulation of 0.25–1 mm, being recommended by the manufacturer for horizontal and vertical bone augmentation, dehiscence, or fenestrations, and for alveolar ridge preservation. The experimental biomaterial is a nanostructured hydroxyapatite reinforced with titanium particles (TiO_2_-HA), which was obtained by a two-stage sintering (TSS) process of a hydroxyapatite powder and a titanium hydride (TiH_2_) powder, HAT 10, named “Bony” [60]. It can be used in tissue engineering, especially for bone regeneration, due to its specific properties, including a homogeneous, densified nanostructure, good biocompatibility, superior wear resistance, and high stability of the hydroxyapatite component [60,61].

### 2.4. Surgical Procedure

**Figure 1:**
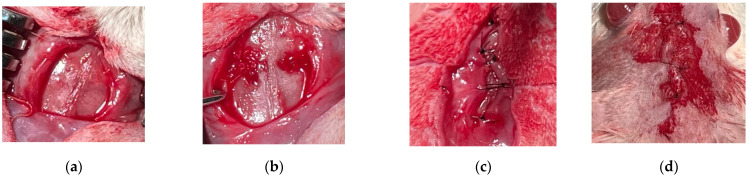
Stages of the surgical procedure: (**a**) Incision with periosteal lift, (**b**) Bone defects created and grafted with biomaterial, (**c**) Periosteal suture, (**d**) Scalp suture.

The surgical procedure began by performing general anesthesia of the laboratory animals with Ketamine 100 mg/mL 20 IU (90 mg/kg) (Ketabel, Bela-pharm GmbH& Co. KG, Vechta, Germany) and Xylazine 2% 0.3 mL (10 mg/kg) (Xylazin Bio, Bio-veta, Czech Republic), supplemented with local anesthesia with Lidocaine 20 mg/mL (4 mg/kg) (Xiline, Zentiva, Prague, Czech Republic). The surgical procedure began by shaving the scalp and antisepticising with Betadine 100 mg/mL (Egis Pharmaceutical, Budapest, Hungary). The bone was discovered after an anteroposterior incision and flap elevation. Subsequently, the bone defect was created with a spherical bur mounted on the surgical piece, continuously irrigated with sterile saline solution. The defects were 3 mm in diameter to allow spontaneous bone healing and to avoid damaging the meninges during the surgical procedure. To determine the exact positions of the defects, the reference line was the midline, with the defects created in the parietal bone on either side of it. After creating the bone defects, the negative control group had an empty defect, the positive control group received bovine xenograft, and the study group received the experimental synthetic material. The surgical procedure was completed by the suture of the periosteum and the scalp with 6-0 and 3-0 sutures, respectively (Supramid, SMI sutures, Sankt Vith, Belgium). To avoid the occurrence of secondary infections, the incisions were covered with betadine (Figure 1).

All surgeries followed the same pre-established protocol, and upon completion, the animals returned to their well-ventilated cages, with a 12 h light/dark cycle and a constant temperature of 25 ± 1 °C. The animals were fed standard food, combined granulated food, and water ad libitum. To avoid postoperative discomfort, the animals were administered subcutaneously with buprenorphine 0.3 mg/mL (0.05 mg/kg) (Buprenex, Hospira, Inc., Lake Forest, IL, USA) analgesics twice daily for 48 h. The healing period was uneventful; no local or systemic complications occurred, and no animals died due to the experiment.

The animals from the three groups were randomly subdivided into subgroups according to the two chosen healing periods, respectively, 2 months and 4 months, as follows:

- Groups A1/A2: negative control group 1/2, in which natural healing was expected, and the animals were sacrificed after 2/4 months.
- Groups B1/B2: positive control group 1/2, in which the bovine xenograft was added, and the animals were sacrificed after 2/4 months.
- Groups C1/C2: study group 1/2, in which the experimental synthetic material based on hydroxyapatite reinforced with titanium particles was grafted, and the animals were sacrificed after 2/4 months.

The samples collected from the formed groups followed a typical evolutionary pattern, with differences observed in the rate of bone neoformation and in the morphological elements observed after 2 and 4 months of healing.

### 2.5. Bone Samples Preparation

The laboratory animals were euthanized after a 2-month or 4-month period, according to their grouping, by anesthetic overdose. The samples containing the defect together with the surrounding tissues were harvested, washed, and fixed by immersion in 10% neutral buffered formalin (Bio-Optica, Milan, Italy) for 48 h.

### 2.6. OCT Analysis

Before being examined with the optical coherence tomography, the samples were washed under a continuous stream of water and carefully dried. OCT was chosen as the method of analysis of the samples because it is a valuable technique for evaluating newly formed bone tissue, offering high spatial resolution and non-invasive detection. The examination was performed using an SSOCT device (THORLABS, OCS1300SS; Munich, Germany) from the University of Medicine and Pharmacy of Craiova. The system has a laser source: a scanning laser (55 kHz), operating at a central wavelength of 1325 nm (average power ≈ 12 mW), enabling two- and three-dimensional scans. The axial resolution was approximately 12 µm, and the lateral resolution was approximately 15 µm. The sample’s optical power was 5 mW. The image configuration used was X-1024 pixels, width 15 mm; Z-512 pixels, width 1.92 mm, contrast 1.8, and brightness 54.

To perform the analysis using OCT, different intensities of the light signal were considered; the method’s disadvantage is the curvature of the sample surface. Thus, to minimize the occurrence of possible errors, the bone samples were positioned so that the analyzed surface was as flat as possible, each sample being fixed in a high-consistency silicone block (Zetaplus L Intro KIT, Zhermack, Badia Polesine, Italy), leaving only the area of interest visible. After completion of the OCT analysis, the samples were reinserted into the 10% formalin tubes.

For each sample analyzed, approximately 600 images were obtained over a 1.6 cm interval. From these, a set of 10 images was selected to cover the bone defect.

### 2.7. Analysis of OCT Images Using Image J Software

The images obtained from OCT analysis of bone samples were analyzed using Image J 1.54p software (National Institutes of Health (NIH), Kensington, Maryland, MD, USA), whose operating principle is based on the possibility of counting pixels in areas of interest, and which quantifies the results in the form of a table showing the integrated pixel density in the selected area of interest. On the OCT image, the higher the porosity of the bone in the analyzed sample, the deeper the penetration of light. The denser the bone, the less light penetrates. Also, the higher concentration of graft particles, the higher brightness in the OCT image, and the higher integrated density.

From the initial set of 10 images, the most representative images were selected: an image from the initial part of the defect, an image from the center of the defect, and an image from the final part of the defect. For each image, three regions of interest (ROI) were selected and analyzed, namely: one from the native bone region, one from the defect area, and one from the newly formed bone region, because the integrated density of a sample is influenced by these bone types [62] and by the refractive index of the graft material particles used [63]. The chosen region of interest was 50 × 50 pixels, with each ROI located in the center of the analyzed area (defect area, newly formed bone area, and native bone area). Since the integrated density in OCT can be strongly influenced by the brightness of the augmentation material, the regions of interest were carefully defined to minimize interpretation bias.

### 2.8. Stereomicroscopic Analysis

Before stereomicroscopic examination, the samples were washed under a continuous stream of water and carefully dried. For microscopic analysis of the samples, the NIKON SMZ 745T stereomicroscope (Nikon Corporation, Tokyo, Japan) of the Faculty of Mechanics of the University of Craiova was used. The samples were analyzed at a maximum magnification of 75× and at a working distance of 115 mm. To perform the analysis under the stereomicroscope, each bone sample was fixed in a high-consistency silicone block (Zetaplus L Intro KIT, Zhermack, Badia Polesine, Italy), leaving only the area of interest visible. This analysis method was chosen for examining the surfaces of the samples. Two-dimensional images were obtained, which were stored in JPG format, allowing for subsequent interpretation.

After completing the stereomicroscopic analysis, the samples were reinserted into the 10% formalin-filled tubes.

### 2.9. Analysis of Stereomicroscopic Images Using Image J Software

Images obtained from the stereomicroscopic evaluation of the samples were analyzed to determine the residual particle size of the biomaterials used for grafting using Image J 1.54p software. The measurement scale was set to 200 µm.

### 2.10. Histological Analysis

For histological analysis, anatomical samples were washed with distilled water and decalcified with 14% ethylenediaminetetraacetic acid (EDTA) solution at 4 °C for 15 days. The decalcification of the bone samples was monitored using a needle, with which the consistency of the samples and the progress of decalcification were constantly checked, until they became sufficiently flexible, without signs of mineralization. Subsequently, the samples were rinsed with phosphate-buffered saline (for 30 min), then with high-purity distilled water (for 30 min), and stored in 70% ethanol at 4 °C (for 2 weeks). After this stage, the samples were embedded in paraffin blocks, and 5 µm sections were cut with a microtome and mounted on glass slides.

For histological analysis, samples were stained with hematoxylin-eosin (HE). The protocol involved deparaffinizing the sections in 4 xylene baths of 60 min each, rehydrating them by immersing them in several baths of ethyl alcohol with decreasing concentration for 4 h, and washing them with running water for 5 s. In the last step, the samples were dehydrated using ethyl alcohol solution for 30 min and clarified by passing them through two baths with xylene solution, for 5 min each. For mounting of the bone samples, Entellan mounting medium (Merck & Co., Inc., Rahway, NJ, USA) was used.

The samples were examined using an Olympus CX 20 microscope (Olympus Corporation, Tokyo, Japan) at the University of Medicine and Pharmacy of Craiova. The images were saved in JPG format to allow for subsequent interpretation.

### 2.11. Analysis of Histologic Images Using Image J Software

Image J 1.54p software and its Bone J plugin were used to analyze the histological images of the bone samples. The parameters studied through this method were the average bone surface area, the average size of the bone trabeculae and the average diameter of the osteocytes.

### 2.12. Statistical Analysis

The obtained data were grouped and analyzed using Microsoft Excel 365 (San Francisco, CA, USA) and the Statistical Package for the Social Sciences (SPSS), version 26 (IBM Corp., Armonk, NY, USA). Continuous variables were reported as minimum and maximum values and mean ± standard deviation (SD). Statistical tests included Shapiro–Wilk’s test and Kolmogorov–Smirnov test for data normality analysis, Levene’s test of equality of variances, Independent *t*-test for group comparisons with the effect size calculated using Cohen’s d, as the mean difference between the groups, divided by the pooled SD, One-Way ANOVA with the effect size denoted as ω^2^, computed based on the Sum of Squares between groups, total Sum of Squares, the degrees of freedom and the Mean Square within the groups (followed by Tukey or Games–Howell post hoc analysis) and Kruskal–Wallis H test with the effect size denoted as η^2^, and computed based on the Kruskal–Wallis statistic, the number of groups and the total sample size (followed by pairwise comparisons performed using Dunn’s procedure, with a Bonferroni correction for multiple comparisons, when statistically significant differences were identified). For this study, the value *p* < 0.05 was considered statistically significant.

## 3. Results

### 3.1. Results of OCT Analysis of Samples

**Figure 2:**
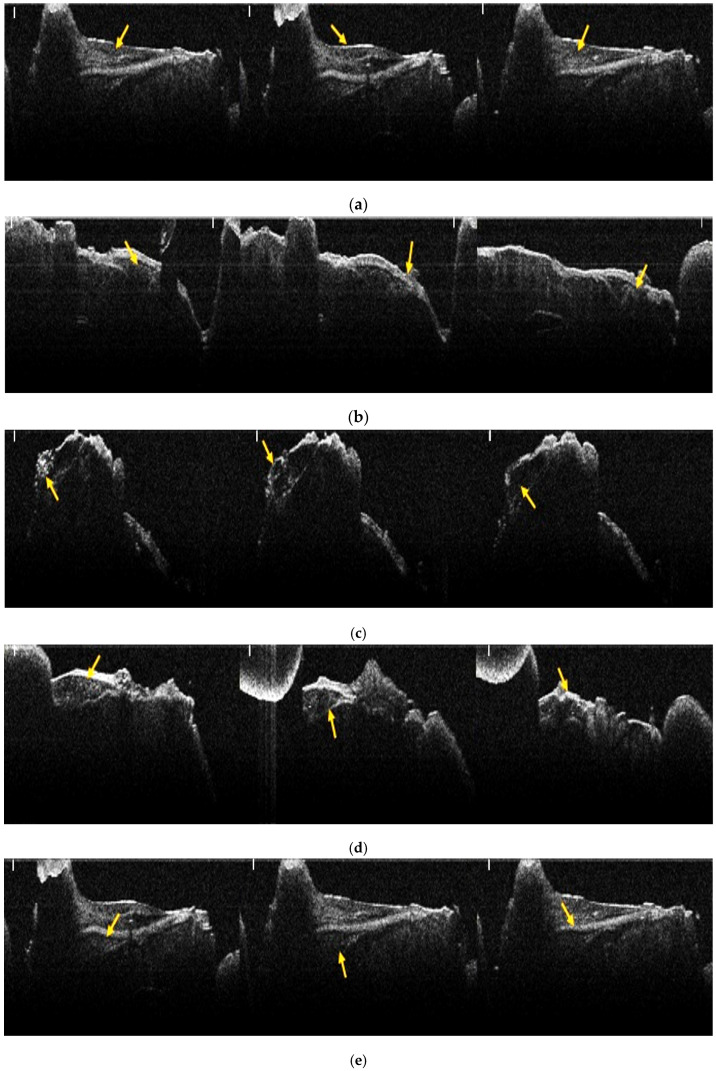

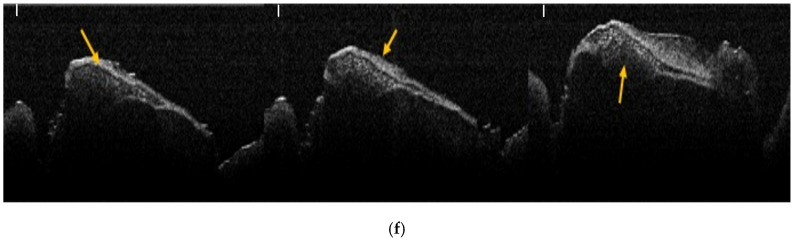
OCT images of bone samples from the three batches: (**a**) Group A1- negative control group- sample collected at 2 months; (**b**) Group A2- negative control group- sample collected at 4 months; (**c**) Group B1- positive control group, to which the xenograft of bovine origin was added- sample collected at 2 months; (**d**) Group B2- positive control group, to which the xenograft of bovine origin was added- sample collected at 4 months; (**e**) Group C1- study group to which the experimental synthetic material was grafted- sample collected at 2 months; (**f**) Group C2- study group to which the experimental synthetic material was grafted- sample collected at 4 months. Yellow arrows mark areas with bone defects (scale bar 100 µm, white line left upper corner).

All samples analyzed included grafted bone, newly formed bone tissue, and native bone (Figure 2). The degree of bone neoformation was evaluated comparatively between the three groups, and between the two follow-up periods, revealing differences in the healing process depending on the type of grafted bone and on natural healing.

### 3.2. Analysis of Bone Evolution Between 2 Months and 4 Months, by Groups and Bone Types

Since the sample groups differed at 2 and 4 months, an independent-samples *t*-test was conducted to determine whether there was a difference in the integrated bone density between 2 and 4 months. No outliers were identified in the data, as boxplot inspection revealed. The Shapiro–Wilk’s test (*p* > 0.05) showed a normal distribution of the integrated density for each group. The Levene’s test for equality of variances (*p* > 0.05) showed homogenous variances.

In the negative control group, there were no statistically significant differences in evolution at 2 months or 4 months, for any of the three bone types (Table 1).

In the positive control group, both native and newly formed bones showed statistically significant differences (*p* < 0.05) between the two time points, with the same pattern: the integrated density was lower at 4 months than at 2 months, indicating a normal evolution. The defect area, however, showed similar values at both time points (Table 1).

In the study group, the integrated density of the native bone was statistically significantly lower at 4 months than at 2 months (*p* = 0.048) (Table 1). However, for the defect area and the newly formed bone, the integrated density was higher at both time points, but without statistical significance.

The variation in integrated density at the level of native bone was different in the three groups between the two time periods, the results showing that in all three groups the values decreased from 2 months to 4 months, the differences being similar between the negative control and study groups, and higher in the positive control group (Table 1). The differences between the two biomaterial groups were statistically significant, unlike those in the natural healing group.

The variation in integrated density at the defect area between 2 months and 4 months showed different values between the three groups. First, in both biomaterial groups, the integrated density in the defect area was twice that in the natural-healing group. The differences between healing at 2 months and 4 months showed a consistent trend in the natural healing groups, a descending trend in density for the bovine xenograft groups, and an increasing trend in density for the study groups (Table 1).

The variation in integrated density in the newly formed bone area showed that in the negative control group, values increased from 2 to 4 months. In the positive control group, the integrated density decreased significantly in 4 months, whereas in the study group, the values increased (Table 1).

### 3.3. Groups Comparisons

For each group and time point, a one-way ANOVA was conducted to determine whether the integrated density differed across the three types of bones included in the study. There were no outliers, as assessed by boxplots; also, the data were normally distributed within each group, as assessed by the Shapiro–Wilk test (*p* > 0.05).

For the negative control group, variances were homogenous, as assessed by Levene’s test of homogeneity of variances for the two moments (*p* > 0.05). Both at 2 months and at 4 months, the mean integrated density was the highest for the defect area, then lower for the newly formed bone, and even lower for the native bone, and the differences between groups were statistically significant, *p* < 0.0005 at 2 months, and *p* = 0.001 at 4 months. The post hoc analysis revealed significant differences between the native bone and the defect area, and between the defect area and the newly formed bone (*p* < 0.05), but not between the other combinations (Table 2).

For the positive control group, there was homogeneity of variances, as assessed by Levene’s test of homogeneity of variances, only at 2 months; thus, Welch ANOVA and the Games-Howell post hoc analysis were run for the integrated density at 4 months. Similarly, Welch ANOVA was run for the study group, both at 2 and 4 months. For both groups, the two-time moments presented statistically significant differences between the different bone types (Table 2). The post hoc analysis revealed that the highest differences were recorded between the defect area and both the native and newly formed bone, being also statistically significantly different. The smallest differences were recorded between the native and newly formed bone, with no statistical significance (Table 2).

In multiple intragroup comparisons, it was observed that bone from the defect area presented a higher integrated density then native bone for all groups, with the largest difference being observed in the study group at 4 months, then in the study group at 2 months, followed by the positive control group, with similar values at 2 months compared to the values at 4 months, being almost half of the difference recorded in the study group. The smallest variation between native bone and bone from the defect area was observed in the negative control group, in which the variation at 4 months was higher than at 2 months. All differences were statistically significant (Table 2).

The comparison between the defect area and the area with newly formed bone, between the two periods (2 months–4 months) showed that the integrated density in the defect area was much higher than the integrated density in the area of newly formed bone in all groups and subgroups, the order of magnitude for the variation being the following: study group at 4 months, study group at 2 months, positive control group at 2 months, positive control group at 4 months (with similar values), negative control group at 2 months and negative control group at 4 months. All differences were statistically significant (Table 2).

The comparison between native bone and newly formed bone, at 2 months and 4 months, showed variable differences between groups and subgroups, in all cases the integrated density had higher values in the case of native bone than in the case of the area with newly formed bone, the order of magnitude being the following: negative control group at 2 months, positive control group at 2 months, study group at 2 months, positive control group at 4 months, negative control group at 4 months and study group at 4 months. All differences were not statistically significant, showing the similarity between native and newly formed bone (Table 2).

### 3.4. Bone Type Analysis

For each bone type and time point, a one-way ANOVA was conducted to determine whether the integrated density differed among the three groups included in the study (negative control, positive control, and study). No outliers were identified in the data, as boxplot inspection revealed. The Shapiro–Wilk’s test (*p* > 0.05) showed a normal distribution of the integrated density for each group. The Levene’s test for equality of variances (*p* > 0.05) showed homogenous variances, for all group comparisons (*p* > 0.05), except for the native bone, at 2 months; thus for this bone type, the Welch ANOVA was used.

Statistically significant differences between the study groups were identified only in the defect area, at 2 and 4 months, and in the newly formed bone, at 2 months. In the defect area, at 2 months, the positive control and the study groups progressed similarly, both being statistically significantly different compared to the negative control group. However, within 4 months, statistically significant differences were observed across all three group combinations, indicating a distinct evolutionary trajectory. For the newly formed bone, evolution was similar between the positive control and study groups, as well as between the negative control and study groups (*p* > 0.05). Also, the integrated density for the positive control group was statistically significantly higher than that for the negative control group (Table 3).

The highest integrated density value was observed in the defect area in the group with the synthetic biomaterial 4 months after its insertion into the rat calvaria, indicating that the synthetic biomaterial did not osseointegrate over this period. Compared to this, the bovine xenograft initiated osteointegration, with an integrated density lower at 4 months than at 2 months. The fact that in the defect area of the negative control group, higher values were recorded than in the native bone area shows that in this area, the bone has not regenerated yet. Compared to the groups in which bone grafts were inserted in the defect area, the integrated density values at this level are much lower. New bone is difficult to evaluate because it is present at the edge of the defect and within the defect, around the grafted particles. The integrated density of the newly formed bone in the graft groups followed the trend of the integrated density values in the defect areas: for bovine graft groups, decreasing from 2 months to 4 months, and for the synthetic biomaterial, increasing from 2 months to 4 months.

### 3.5. Results of Stereomicroscopic Analysis of Samples

The images of the bone samples obtained from the stereomicroscopic evaluation were analyzed using Image J software. In these images, the stage of bone defect healing was highlighted, as well as the presence of residual biomaterial particles.

**Figure 3:**
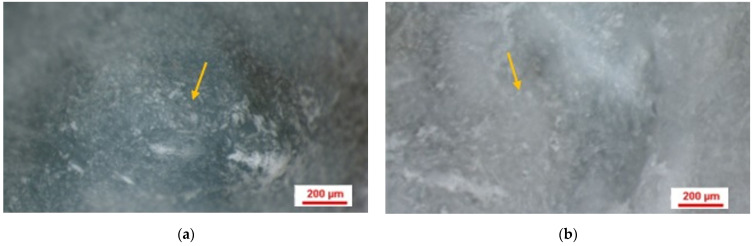

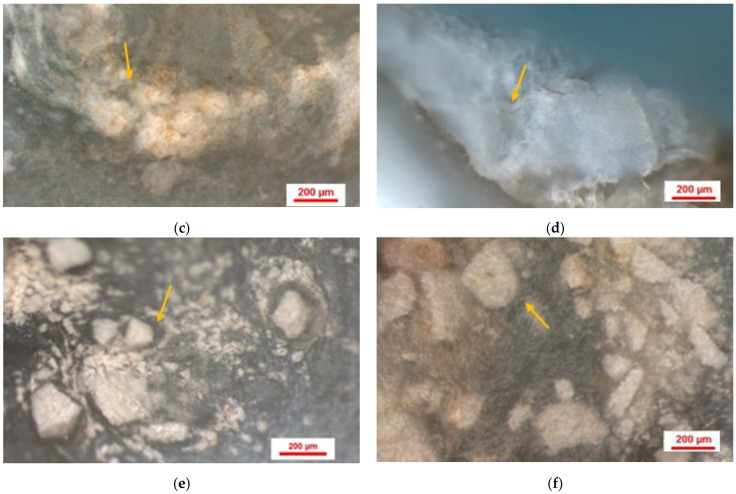
Stereomicroscopic images with 75x magnification. (**a**) Negative control group- natural healing- sample collected at 2 months (**b**) Negative control group- natural healing- sample collected at 4 months (**c**) Positive control group- bovine xenograft- sample collected at 2 months (**d**) Positive control group- bovine xenograft- sample collected at 4 months (**e**) Study group- synthetic nanocomposite based on hydroxyapatite reinforced with titanium particles- sample collected at 2 months (**f**) Study group- synthetic nanocomposite based on hydroxyapatite reinforced with titanium particles- sample collected at 4 months (yellow arrow marks the area with newly formed bone tissue).

In the negative control group with natural healing, stereomicroscopic images revealed dark areas containing new bone tissue (Figure 3a,b). In the stereomicroscopic pictures of the positive control group, in which a bovine xenograft was added (Figure 3c,d), the presence of residual biomaterial particles was observed, both at 2 months and at 4 months. On the stereomicroscopic images from 2 months (Figure 3c), newly formed bone tissue could be observed surrounding the residual biomaterial particles that had an average diameter of 0.502 mm (Table 4). In the stereomicroscopic images at 4 months (Figure 3d), newly formed bone evolved into native bone, and the average diameter of the residual biomaterial particles decreased to 0.313 mm (Table 4), with a more homogeneous structure. In the stereomicroscopic images corresponding to the study group (Figure 3e,f), in which the synthetic nanocomposite based on hydroxyapatite reinforced with titanium particles was grafted, it was noted that at 2 months (Figure 3e) the residual particles had an average diameter of 0.403 mm (Table 4), with a clear outline of the newly formed bone tissue being observed around them. At 4 months, the residual particles had an average diameter of 0.396 mm (Table 4). Their number decreased, but not significantly, and the structure was more homogeneous.

The mean diameter of the residual biomaterial particles decreased between the two healing periods, with a statistically significant difference observed in the bovine xenograft group (*p* = 0.042). The comparison between groups at 2 months showed that in the negative control group the mean residual particle diameter was larger than in the study group, but the difference was not statistically significant (*p* = 0.306) (Table 4). At 4 months, the mean diameter of the residual particles was larger in the study group. Still, the difference was not statistically significant (*p* = 0.066) (Table 4).

However, stereomicroscopic images showed a higher and more rapid degree of transformation into new bone tissue in the bovine xenograft than in the titanium reinforced hydroxyapatite synthetic nanocomposite (Table 4).

### 3.6. Results of Histological Analysis of Samples

**Figure 4:**
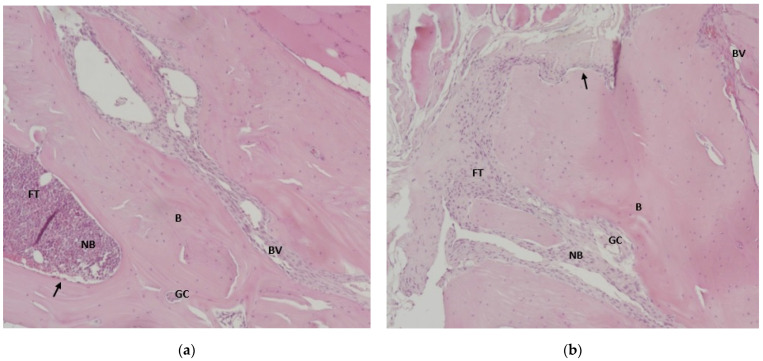

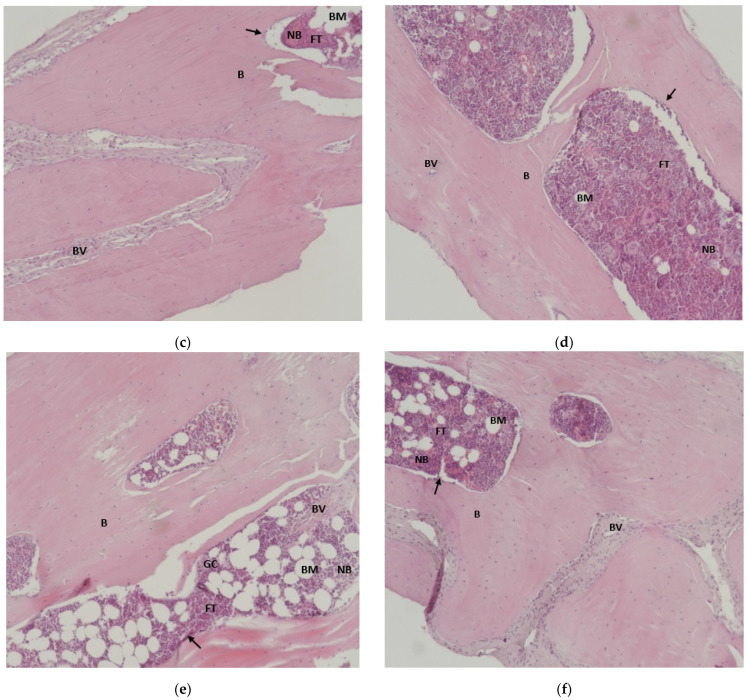
Hematoxylin-eosin-stained bone defect samples. (**a**) Negative control group at 2 months (**b**) Negative control group at 4 months (**c**) Positive control group (bovine xenograft) at 2 months (**d**) Positive control group (bovine xenograft) at 4 months (**e**) Study group (synthetic biomaterial) at 2 months (**f**) Study group (synthetic biomaterial) at 4 months. Legend of abbreviations: BM—biomaterial, NB—newly formed bone, B—native bone, FT—fibrous tissue, BV—blood vessel, GC—giant cells, black arrow (↑)—indicates the row of osteoblasts that delimit the areas of newly formed bone. (**a**) Negative control group (natural healing) at 2 months—trabecular bone, with areas of young bone alternating with lamellar areas, in which periosteal remodeling is visible (**b**) Negative control group (natural healing) at 4 months—lamellar trabecular bone with hematogenous marrow, with megakaryocyte giant cells and with confluent areas of endosteal remodeling (**c**) Positive control group (bovine xenograft) at 2 months—trabecular bone with areas of endo-periosteal bone remodeling and areas of active bone formation (**d**) Positive control group (bovine xenograft) at 4 months—lamellar trabecular bone, with an area of intramatrix bone remodeling, with the presence of hematogenous marrow with giant megakaryocyte cells (**e**) Study group (synthetic biomaterial) at 2 months—Microscopic structure of lamellar trabecular bone with disordered arrangement of osteocytes, showing intratrabecular hematogenous bone marrow and fine areas of periosteal and endosteal bone remodeling (**f**) Study group (synthetic hydroxyapatite reinforced with titanium particles) at 4 months—Cancellous lamellar bone with large areas of periosteal bone remodeling (Magnification 10×).

In the images from the negative control group, extensive areas of native bone were observed at 2 and 4 months (Figure 4a,b). Blood vessels were noted in the areas of bone defect, and osteoblast lamellae were observed in the areas of newly formed bone tissue (Figure 4a,b). In histological images of the positive control group at both 2 and 4 months, areas of native bone with osteocyte lacunae were observed near areas containing particles of grafted biomaterial, surrounded by osteoblast lamellae and newly formed bone tissue (Figure 4c,d). In the study group, at both 2 and 4 months, areas of native bone were observed, with newly formed bone tissue and osteoblast lamellae surrounding the particles of the grafted biomaterial, which remained mainly unchanged (Figure 4e,f).

Bone image analysis using the Image J plug-in Bone J was interpreted in terms of bone surface, trabecular thickness, and osteocyte diameter.

An independent *t*-test was used to determine whether there was a statistically significant mean difference between the 2-month and 4-month follow-ups, for all three study groups, for all three parameters: bone surface, thickness of the trabeculae, and diameter of the osteocytes. Although the analysis detected an outlier that was more than 1.5 box lengths from the edge of the box in a boxplot, it was not extreme, so we decided to keep it for the further analysis. Shapiro–Wilk’s test (for smaller datasets) and Kolmogorov–Smirnov (for large datasets) (*p* > 0.05) were used to assess its value. The results showed that the assumption of normality was not violated.

The bone surface was similar between 2 and 4 months, for all three groups, without any statistically significant differences (*p* > 0.05). The mean thickness of bone trabeculae decreased after 4 months compared with the 2-month follow-up in all three groups. Still, the differences were statistically significant only for the negative control and study groups (*p* < 0.05). The mean osteocyte diameter also decreased after 4 months compared with the 2-month follow-up for all three groups. Still, the differences were statistically significant only for the study groups (*p* < 0.0005) (Table 5).

A Kruskal–Wallis test was conducted to determine if there were differences in the histological parameters between the three study groups, both for 2-month and 4-month follow-ups (Table 5). Distributions of parameters were similar for all groups, as assessed by visual inspection of a boxplot. The bone surface was not statistically significantly different between the three other groups at 2 months (χ^2^(2) = 0.974, *p* = 0.614) and at 4 months (χ^2^(2) = 2.213, *p* = 0.331). Although not statistically significant, the values were different between the three groups, being the highest in the negative control group at 2 months, followed by the positive control group at 4 months, the positive control group at 2 months, the negative control group at 4 months, the study group at 2 months, and the last being the study group at 4 months.

On the other hand, the mean thickness of the trabeculae was statistically significantly different between the different groups, at 2 months with χ^2^(2) = 411.238, *p* < 0.0005, as well as at 4 months with χ^2^(2) = 1016.438, *p* < 0.0005 (Table 5). For pairwise comparisons, Dunn’s procedure was used. For multiple comparisons, Bonferroni correction with a level of *p* < 0.0166 of statistical significance was used. For the 2-month follow-up, this post hoc analysis revealed statistically significant differences in mean trabecular thickness between the negative control and positive control groups (*p* < 0.0005) and between the negative control and study groups (*p* < 0.0005), but not between any other group combinations.

Also, the mean osteocyte diameter was statistically significantly different between groups, at 2 months with χ^2^(2) = 14.348, *p* = 0.001, but not at 4 months with χ^2^(2) = 1.159, *p* = 0.560 (Table 5). For pairwise comparisons, Dunn’s procedure was used. For multiple comparisons, Bonferroni correction with a level of *p* < 0.0166 of statistical significance was used. The post hoc analysis at 2-month follow up showed a statistically significant differences in the mean osteocyte diameter between the study and positive control groups (*p* = 0.008) and between the study and negative control groups (*p* = 0.001), but not between the positive and negative control groups.

## 4. Discussion

The present study was conducted on the calvaria of adult male Wistar rats to compare the degree of osteointegration of two biomaterials, a bovine xenograft and a synthetic nanocomposite based on hydroxyapatite reinforced with titanium particles. The study showed that the bovine graft material integrated better than the synthetic one, with the size of the biomaterial particles significantly decreasing between 2 and 4 months in the bovine xenograft group. In contrast, in the synthetic material group, it remained almost the same. An important reason for this phenomenon is the titanium reinforcement of the synthetic material particles [60,61]. Thus, both biomaterials are suitable scaffolds for biological materials to develop, but in principle, the synthetic biomaterial has a longer osteointegration period [43]. The use of these biomaterials for alveolar ridge preservation remains questionable [33].

In contrast with the bovine xenograft that integrated partially, the synthetic material with an irregular granulation and the particles initially smaller compared to the xenograft, remained at almost the same size over the 4 months.

The present study used several experimental methods to analyze osteointegration of biomaterial particles in the rat calvaria: optical coherence tomography, stereomicroscopy, and hematoxylin and eosin histological staining.

Optical coherence tomography, with its advantages of non-invasiveness, ease of use, radiation-free operation, and non-ionizing nature, is a valuable method for analyzing bone formation in defects created in the rat calvaria [49,62,64,65,66]. Two-dimensional cross-sectional OCT images highlight areas of bone healing in different shades of gray. OCT images were analyzed using Image J software, which allows counting pixels in areas of interest and calculating integrated density values. In the study conducted by Khaddour et al., the degree of osseointegration of two biomaterials, a porcine xenograft and a synthetic biomaterial based on hydroxyapatite reinforced with titanium particles, was evaluated, and the results were analyzed at 8 and 16 weeks. OCT examination showed that the porcine xenograft tended to transform into native bone more quickly. In contrast, the synthetic biomaterial delayed healing, resulting in a slower rate of new bone formation [4]. In the study by Rădoi et al., OCT was used to assess the degree of bone formation in defects grafted with two synthetic hydroxyapatite-based materials. Their results showed variability in the healing pattern, especially in the persistence of some areas of the defect in which new bone tissue was not formed [67]. In the present study, the comparison between native bone, newly formed bone, and bone in the defect area showed that the integrated density was higher in the defect area. Integrated density in the defect area was twice as high in the biomaterials groups as in the natural-healing group, which is explained by the presence of the bone graft particles in that area.

Bovine bone particles that are highly mineralized are distinguished by the brightest areas in the OCT images [62]. Similarly, synthetic biomaterial particles reinforced with titanium appear bright in the OCT image. The higher the integrated density, the higher the degree of mineralization of the bone. In the case of grafted bone particles, this means that they have not been osseointegrated, the native bone having a lower integrated density than the grafted bone. As Luca et al. [62] argue, the brightness level is an indicator of bone density. This information allows the calculation of trabecular mineralization and indicates the direction and speed of bone growth [68].

Stereomicroscopy was used to evaluate rat calvaria bone fragments in another study that compared porcine bone and titanium-reinforced hydroxyapatite biomaterials with natural healing. The study showed that the porcine xenograft was incorporated more rapidly into the native bone, whereas the synthetic biomaterial based on titanium-reinforced hydroxyapatite particles required a longer period [43]. Thus, in our study, as in the study by Khaddour et al. [43], it was observed that the bovine xenograft particles integrated more rapidly than those of the synthetic biomaterial, which remained the same size and were clearly visible on the stereomicroscopy images.

Histological analysis of the samples confirmed what the stereomicroscopy and OCT images showed: the bovine xenograft elicited a better tissue response than the synthetic biomaterial, with better incorporation into the bone tissue at 4 months. The bone surface images analyzed with Image J showed a similarity between the samples grafted with bovine bone and those with natural healing, while the bone surface in the samples with the synthetic biomaterial was much reduced at both 2 months and 4 months. In the analyzed images, the thickness of the bone trabeculae was significantly greater in grafted bones, both with xenograft and with synthetic biomaterial, compared to the bone with natural healing. The average osteocyte diameter was significantly different between the three groups at 2 months, with the smallest in the natural-healing group, followed by the bone with synthetic biomaterial, and then the bone with bovine xenograft. At 4 months, the differences were not significant. However, osteocyte diameter varied, with the smallest in the synthetic biomaterial group, followed by the natural-healing group, and then the bovine xenograft group. The study by Bae et al. aimed to evaluate bone regeneration in defects grafted with porcine and bovine xenografts at 4 and 8 weeks. The results for the new bone surface did not show significant differences between porcine (25.22 ± 13.56%) and bovine (21.68 ± 11.11%) xenografts at 8 weeks [69].

If animal xenografts or synthetic hydroxyapatite reinforced with titanium particles are used as grafting biomaterials, it is essential to consider the long-term risks. In the literature, it has been highlighted that these types of biomaterials can elicit persistent inflammatory responses, incomplete osseointegration, immune reactions, and the formation of fibrous tissue at the biomaterial-native bone interface [32]. In a study by Bielenstein et al., a bovine xenograft and a synthetic biomaterial were compared. The results showed a higher inflammatory response in the synthetic material group and a higher anti-inflammatory reactivity in the bovine xenograft group [70]. In the study by Drăghici et al., the biocompatibility and osteointegration of the experimental synthetic material, a hydroxyapatite reinforced with titanium particles, were compared with those of a commercially available synthetic biomaterial based on hydroxyapatite and beta-tricalcium phosphate. Their results showed that the experimental material elicited an initially more intense inflammatory reaction, followed by the appearance of dense but less uniform bone tissue, with more residual particles than in the commercially available synthetic bone [52]. In the study by Kim et al., natural healing, the bovine xenograft we also used in the present study, and a hydroxyapatite-based biomaterial were compared, and the results were analyzed at 4 and 8 weeks. They concluded that at 8 weeks, there were no statistically significant differences among the three groups in bone neoformation, with the hydroxyapatite-based biomaterial showing results similar to those of the bovine xenograft [55]. A study compared the degree of bone neoformation in bone defects grafted with autogenous bone, Bio-Oss, Emdogain, or a combination of BoneCeramic and Emdogain, at 30 and 60 days. They reported a higher percentage of new bone tissue formation in the autogenous bone group than in all other study groups [53].

Other new biomaterials based on hydrogels are currently studied. For example, in the study by Guo et al., a 3D biomaterial based on a photocurable polyether F127 diacrylate hydrogel loaded with mixed spheroids of mesenchymal stem cells and vascular endothelial cells was analyzed. They demonstrated the osteogenic and angiogenic potential of the biomaterial, which promoted bone repair and alveolar ridge preservation [54].

The results of the present study showed that there were statistically significant differences in bone healing between the presence of biomaterials and natural healing, thereby contradicting the null hypothesis.

A limitation of the study was that the bone samples came from different rats, leading to variability in the healing process among animals. Also, the extrapolation of the results to humans is a limitation. Another limitation may be the influence of the integrated density measured on the OCT image by the brightness of the augmentation material. To address this limitation, strict measures were taken to select regions of interest for analysis in Image J. The study samples are indeed limited to 5 in each subgroup; however, this value is acceptable for the statistical test performed within this study.

## 5. Conclusions

The study showed that the two biomaterials (bovine xenograft and a synthetic hydroxyapatite reinforced with titanium) did not entirely integrate at 4 months. The bovine xenograft, due to its porous structure and organic composition, had a more favorable healing pattern than the synthetic nanocomposite based on hydroxyapatite reinforced with titanium particles, which, due to its slow resorption capacity, can provide prolonged structural stability. Neither of the two biomaterials are indicated for ARP. Therefore, the selection of the material used for grafting bone defects must be based on the requirements and specifics of each case. Further studies are needed to evaluate the properties of the new biomaterial, as well as clinical trials to assess its effectiveness in human subjects.

## Figures and Tables

**Table 1:** Comparative analysis of the integrated density values for the three groups at 8 and 16 weeks, depending on bone area.

Sample		Integrated Density									
		2 Months			4 Months			Differences (2 Months–4 Months)			
		Min	Max	Mean ± SD	Min	Max	Mean ± SD	Mean ± SD	*p* */ Cohen’s d	95% CI Lower	95% CI Upper
Negative control group	Native bone	13.36	23.52	19.878 ± 4.297	9.22	20.58	16.012 ± 4.091	3.866 ± 3.769	0.141/ 0.992	−1.530	9.262
	Defect area	28.87	41.24	33.539 ± 4.61	17.23	40.03	32.849 ± 8.63	0.691 ± 9.266	0.866/ 0.100	−8.209	9.590
	Newly formed bone	8.62	20.78	15.369 ± 4.818	12.43	24.86	19.689 ± 4.885	−4.32 ± 7.633	0.154/ 0.890	−10.560	1.920
Positive control group	Native bone	15.95	35.05	25.48 ± 8.172	13.49	22.03	16.493 ± 3.064	8.987 ± 7.534	0.043 ^#^/ 1.456	0.392	17.580
	Defect area	46.18	78.72	59.382 ± 12.322	27.97	71.91	52.33 ± 15.979	7.053 ± 12.988	0.412/ 0.494	−11.301	25.407
	Newly formed bone	21.82	28.75	25.183 ± 3.065	14.9	27.69	18.583 ± 5.299	6.6 ± 7.791	0.025 ^#^/ 1.525	1.031	12.168
Study group	Native bone	12.66	23.6	17.66 ± 3.643	11.55	15.92	13.979 ± 1.645	3.681 ± 4.803	0.048 ^#^/ 1.302	0.045	7.317
	Defect area	50.2	85.87	65.442 ± 14.967	52.56	90.21	74.583 ± 12.792	−9.141 ± 19.433	0.282/ 0.657	−27.050	8.769
	Newly formed bone	11.58	25.07	19.698 ± 4.622	15.59	32.86	21.852 ± 5.786	−2.154 ± 7.497	0.493/ 0.411	−8.890	4.582

* Independent *t*-test, comparative analysis within the groups at 2 and 4 months, according to the bone area. ^#^ Statistically significant.

**Table 2:** Integrated density differences among the various bone types, for each study group—ANOVA result and post hoc group comparisons.

Group	Bone Type	2 Months		4 Months	
Negative control group	ANOVA/*p*/ω^2^	(F(2, 15)) 25.608/<0.0005 ^#^, ω^2^ = 0.732		(F(2, 15)) 12.258/0.001 ^#^, ω^2^ = 0.556	
	Multiple group comparisons	Variation	*p* **	Variation	*p* *
	Native—Defect area	−13.661	<0.0005	−16.837	0.001
	Defect area—Newly formed	18.170	<0.0005	13.159	0.006
	Native—Newly formed	4.509	0.236	−3.677	0.571
Positive control group	ANOVA/*p*/ω^2^	(F(2, 15)) 30.515/<0.0005 ^#^, ω^2^ = 0.766		(F(2, 8.375)) 13.531/0.002 ^##^, ω^2^ = 0.726	
	Multiple group comparisons	Variation	*p* *	Variation	*p* **
	Native—Defect area	−33.902	<0.0005	−35.836	0.006
	Defect area—Newly formed	34.199	<0.0005	33.746	0.006
	Native—Newly formed	0.297	0.998	−2.089	0.693
Study group	ANOVA/*p*/ω^2^	(F(2, 8.936)) 26.894/<0.0005 ^##^, ω^2^ = 0.462		(F(2, 15)) 97.811/<0.0005 ^#^, ω^2^ = 0.915	
	Multiple group comparisons	Variation	*p* **	Variation	*p* **
	Native—Defect area	−47.782	0.001	−60.604	<0.0005
	Defect area—Newly formed	45.743	0.001	52.730	<0.0005
	Native—Newly formed	−2.038	0.684	−7.873	0.248

^#^ One-way ANOVA test. ^##^ Welch’s ANOVA test. * Tukey post hoc analysis. ** Games–Howell post hoc analysis.

**Table 3:** For each type of bone, the overall result and multiple group comparisons- integrated density variation between different study groups.

Bone Type	Group	2 Months		4 Months	
Native bone	ANOVA/*p*/ω^2^	(F(2, 9.368)) 2.220/0.162 ^##^, ω^2^ = 0.179		(F(2, 15)) 1.112/0.355 ^#^, ω^2^ = 0.012	
	Multiple group comparisons	Variation	*p* **	Variation	*p* *
	Negative control—Positive control	-	-	-	-
	Positive control—Study	-	-	-	-
	Negative control—Study	-	-	-	-
Defect area	ANOVA/*p*/ω^2^	(F(2, 15)) 13.013/0.001 ^#^, ω^2^ = 0.572		(F(2, 15)) 15.907/<0.0005 ^#^, ω^2^ = 0.624	
	Multiple group comparisons	Variation	*p* *	Variation	*p* *
	Negative control—Positive control	−25.843	0.004	−19.481	0.047
	Positive control—Study	−6.059	0.641	−22.253	0.023
	Negative control—Study	−31.902	0.001	−41.734	< 0.0005
Newly formed bone	ANOVA/*p*/ω^2^	(F(2, 15)) 8.068/0.004 ^#^, ω^2^ = 0.440		(F(2, 15)) 0.583/0.571 ^#^, ω^2^ = 0.049	
	Multiple group comparisons	Variation	*p* *	Variation	*p* *
	Negative control—Positive control	−9.814	0.003	-	-
	Positive control—Study	5.485	0.097	-	-
	Negative control—Study	−4.329	0.214	-	-

^#^ One-way ANOVA test. ^##^ Welch’s ANOVA test. * Tukey post hoc analysis. ** Games–Howell post hoc analysis.

**Table 4:** Measurements of residual biomaterial particles on stereomicroscopic images between study groups in the two follow-up periods.

Sample	Measurements				*p* (2M-4M)/Cohen’s d
	2 Months		4 Months		
	Mean	Standard Deviation	Mean	Standard Deviation	
Positive Control Group (Bovine Xenograft)	0.502	0.156	0.313	0.059	0.042 *^,#^/1.316
Study Group (Synthetic Hydroxyapatite reinforced with Titanium particles)	0.403	0.092	0.396	0.083	0.894 */ 0.186
*p*/Cohen’s d	0.306 **/0.523		0.066 **/1.159		

* comparative analysis within the group at 2 months and 4 months, *t*-test. ** comparative analysis between the two materials, at 2 months and 4 months, *t*-test. ^#^ Statistically significant, *p* < 0.05.

**Table 5:** Histological analysis of samples from the three groups during the 2 follow-up periods.

Sample		Bone Surface			Mean Thickness of the Trabeculae			Mean Diameter of the Osteocytes		
		2 Months	4 Months	*p* */d	2 Months	4 Months	*p* *	2 Months	4 Months	*p* */d
Negative Control	Mean ± SD	85.972 ± 120.942	60.25 ± 94.353	0.717/0.237	7.270 ± 25.371	6.209 ± 24.435	0.030 ^#^/0.043	14.53 ± 40.607	13.965 ± 41.768	0.679/0.014
	Median	35.970	7.650		2.734	2.734		8.816	8.201	
Positive Control	Mean ± SD	75.467 ± 105.102	82.387 ± 112.114	0.922/0.064	13.788 ± 46.655	11.751 ± 49.391	0.349/0.042	20.196 ± 58.600	16.034 ± 62.617	0.242/0.069
	Median	35.970	40.230		5.114	6.234		7.128	7.828	
Study Group	Mean ± SD	43.887 ± 70.225	35.752 ± 57.168	0.846/0.127	12.939 ± 32.158	10.349 ± 23.597	0.010 ^#^/0.092	18.116 ± 39.244	11.589 ± 29.054	<0.0005 ^#^/0.189
	Median	6.123	5.210		6.583	7.732		8.201	7.828	
*p* **/η^2^		0.614/ 0.002	0.331/0.093		<0.0005 ^#^/0.053	<0.0005 ^#^/0.131		0.001 ^#^/0.004	0.560/0.001	

* comparative analysis within the group at 2 months and 4 months, Independent *t*-test. ** comparative analysis between the three materials, at 2 months and 4 months, Kruskal–Wallis H test. ^#^ Statistically significant, *p* < 0.05.

## Data Availability

The original contributions presented in the study are included in the article, further inquiries can be directed to the corresponding author.

## Abbreviations

The following abbreviations are used in this manuscript:

ARP	Alveolar ridge preservation
OCT	Optical coherence tomography
TSS	Two- stage sintering
IU	International unit
NIH	National Institutes of Health
ROI	Region of interest
EDTA	ethylenediaminetetraacetic acid
HE	Hematoxylin-eosin
SPSS	Statistical Package for Social Sciences
SD	Standard deviation
